# Biological Control and Plant Growth Promotion by Volatile Organic Compounds of *Trichoderma koningiopsis* T-51

**DOI:** 10.3390/jof8020131

**Published:** 2022-01-28

**Authors:** Jiaqi You, Guoqing Li, Chaohan Li, Lihua Zhu, Hongjuan Yang, Ronghao Song, Weihong Gu

**Affiliations:** 1 Shanghai Key Lab of Protected Horticultural Technology, Horticultural Research Institute, Shanghai Academy of Agricultural Sciences, Shanghai 201106, China; youjiaqi@saas.sh.cn (J.Y.); lch505@163.com (C.L.); zhulihua.007@163.com (L.Z.); yanghj681114@163.com (H.Y.); 2 State Key Laboratory of Agricultural Microbiology, Collage of Plant Science and Technology, Huazhong Agricultural University, Wuhan 430070, China; guoqingli@mail.hzau.edu.cn; 3 Plant Protection Research Institute, Shanghai Academy of Agricultural Science, Shanghai 201106, China; rhsong15@163.com

**Keywords:** biological control, trichoderma, VOCs, *botrytis cinerea*, *fusarium oxysporum*

## Abstract

*Trichoderma* spp. are widely used in plant disease control and growth promotion due to their high efficacy and multiple biocontrol mechanisms. *Trichoderma koningiopsis* T-51 is an effective biocontrol agent against gray mold disease by direct contact. However, the indirect physical contact biocontrol potential of *Trichoderma* spp. is not clear. In this study, the volatile organic compounds (VOCs) produced by T-51 showed high inhibitory activity against plant pathogenic fungi *Botrytis cinerea* and *Fusarium oxysporum*. The percentage of *B. cinerea* and *F. oxysporum* mycelial growth inhibition by T-51 VOCs was 73.78% and 43.68%, respectively. In both *B. cinerea* and *F. oxysporum*, conidial germination was delayed, and germ tube elongation was suppressed when exposed to T-51 VOCs, and the final conidial germination rate of *B. cinerea* decreased significantly after T-51 treatment. The VOCs from T-51 reduced the *Botrytis* fruit rot of tomato compared with that noted when using the control. Moreover, the T-51 VOCs significantly increased the size and weight of *Arabidopsis thaliana* seedlings. Twenty-four possible compounds, which were identified as alkenes, alkanes, and esters, were detected in VOCs of T-51. These results indicate that *T. koningiopsis* T-51 can exert biological control by integrating actions to suppress plant disease and promote plant growth.

## 1. Introduction

Volatile organic compounds (VOCs) are carbon-based small chemicals with low molecular weight (<300 Da), low polarity, and high vapor pressure (approximately 0.01 kPa) at room temperatures [1,2,3]. VOCs, which are widely produced by microbes, are ideal info-chemicals because of their ability for long-distance diffusion and communication between different living organisms, such as microbe–microbe, or plant–microbe interactions [4,5]. 

The VOCs from *Trichoderma* spp. have been widely reported for their antibiotic and plant growth promotion ability. For example, 6-pentyl-2H-pyran-2-one, which was first characterized from *T. viride* [6] and later from other species such as *T. harzianum* [7] and *T. koningii* [8], showed high antifungal activity against plant pathogenic fungi such as *Botrytis cinerea*, *Fusarium oxysporum*, and *Rhizoctonia solani* [9]. 

*B. cinerea* Per.: Fr. is a cosmopolitan plant pathogenic fungus, which can infect more than 1400 plant varieties [10,11], such as tomato, strawberry, grape, etc. *B. cinerea* is a typical necrotrophic plant pathogen that often causes pre- and postharvest gray mold disease. *F. oxysporum* is a well-known soilborne pathogenic fungus with a very broad host range [12]. *Trichoderma* spp. have been successfully used as biological controls for plant diseases including gray mold and Fusarium wilt diseases [13].

*T. koningiopsis* isolate T-51 is a soilborne fungus that showed excellent biological control and plant growth-promoting potential in our previous study [14]. The interactions between T-51 and *B. cinerea* (mycoparasitism) as well as T-51 and tomato plant (growth promotion and induced resistance) were described based on direct physical contact. However, the indirect (non-physical) effects of T-51 on pathogen and plant were still unknown. In this study, the VOCs produced by T-51 were analyzed and evaluated for the inhibition of plant pathogens *B. cinerea* and *F. oxysporum*, growth promotion of *Arabidopsis thaliana*, and postharvest biocontrol of tomato gray mold. The study aimed to determine whether VOCs of T-51 acted as a long-distance biocontrol factor despite T-51 not being in contact with the pathogen or plant.

## 2. Materials and Methods

### 2.1. Fungal Isolates and Growth Condition

The four *Trichoderma* isolates (*T. koningiopsis* T-51, *T. koningiopsis* T-35, *T. harzianum* T-21 and *T. harzianum* T-68) used in this study were isolated from agricultural soil and were identified in a previous study [14]. The phytopathogenic fungus *B. cinerea* RoseBc-3 was isolated from a flower of Chinese rose showing gray mold disease [14]; the *F. oxysporum* isolate Fon2019 was isolated from the base stem of a watermelon showing Fusarium wilt disease and identified by sequencing blast after PCR using ITS1/ITS4 primer pair described as in previous study [14]. All fungal isolates were stored as mycelial plugs in 20% (*v/v*) glycerol solution at −80 °C. Before experimental use, the fungal isolates were cultured on potato dextrose agar (PDA, Yuanye Bio-Technology Co., Ltd., Shanghai, China) plates at 20 °C and were plated at least twice until the growth rate recovered.

### 2.2. Antifungal Activity of the VOCs of Trichoderma Isolates

The antifungal activity of the VOCs produced by *Trichoderma* isolates was first tested using a double-dish method as previously described [15,16,17]. *Trichoderma* isolates were grown on PDA plates for 48 h for the double-dish setup. In the mycelial growth inhibition experiment, a PDA plate inoculated with *B. cinerea* was placed over a PDA plate inoculated with *Trichoderma*, facing each other and then wrapped with parafilm. A new PDA plate, instead of a *Trichoderma* plate, was used as a control. The double-dish sets were placed at 20 °C for *B. cinerea*. The colony diameter of *B. cinerea* and *F. oxysporum* was measured when the colony in the control plate just reached the edge of the plate. From the results obtained in the first test, T-51 VOCs showed the best inhibition against *B. cinerea*, only T-51 was used in the next studies. The antifungal activity of the VOCs produced by T-51 against *F. oxysporum* mycelial growth were processed as *B. cinerea*, but placed at 28 °C for better growth.

To observe the morphological characteristics of *B. cinerea* and *F. oxysporum* hyphae after treatment with the T-51 VOCs, a double-dish setup using *B. cinerea* or *F. oxysporum* on a cellophane film was placed on a PDA plate (upside) and a T-51 plate, which was grown for 48 h and was placed (downside) facing the bottom plate. The control was set as a double dish using a PDA plate without T-51. The plates were incubated at 20 °C (*B. cinerea*) or 28 °C (*F. oxysporum*) for 3 days, the colony margin of *B. cinerea* and *F. oxysporum* with cellophane film was cut into small pieces (5 mm × 5 mm), fixed immediately in 2.5% glutaral at 4 °C overnight, then dehydrated, critical point dried, gold-coated and examined under a scanning electron microscope (SU8010, Hitachi, Tokyo, Japan) using the standard procedures [17].

In the conidial germination experiment, the conidia of *B. cinerea* and *F. oxysporum* were washed from a 10-day-old colony on PDA plate, filtered and diluted to 1 ×10^5^ conidia/mL using sterilized water. To provide a clear obversion under the microscope, water agar (15 g/L of agar) was used for culture *B. cinerea* and *F. oxysporum* spores in this assay. A double dish of plant pathogenic fungi and T-51 was set using 100 μL of *B. cinerea* or *F. oxysporum* conidia suspension on water agar plate (upside) and *Trichoderma* isolate T-51 on PDA plate (downside). A double-dish setup of *B. cinerea* or *F. oxysporum* and a blank PDA plate was used as a control. The double-dishes were incubated at 20 °C (*B. cinerea*) or 28 °C (*F. oxysporum*) in the dark. Both treatment and control had 12 plates replications. At 3, 6, 9, and 12 h post inoculation, three plates from treatment and control were taken out to check the conidia germination and germ tube length of plant pathogenic fungi under a light compound microscope. At least 150 conidia were counted for germination and 30 germ tubes were rapidly measured in every plate.

### 2.3. Efficacy of VOCs of T-51 in Control of B. Cinerea Fruit Rot of Tomato

The bioassay was proceeded in an airtight glass desiccator (diameter × height = 18 × 23 cm, approximately 5.8 L in volume) as described previously [16]. At the bottom of the desiccator were, 2, 3, 4, or 5 of T-51 PDA plates (48 h old colony) without cover. Healthy red cherry tomato fruits (3.5–4.0 × 2.5–3.0 cm, longitudinal × transverse diameters) bought from a market in Wuhan, China, were washed using tap water, then surface sterilized in 70% ethanol for 3 s, washed in sterile distilled water twice, and airdried. Five tomatoes were put in a clean plate, wounded using a sterilized scalpel, and individually inoculated with 10 μL conidial suspension of *B. cinerea* (1×10^6^ conidia/mL). The plate containing inoculated tomato was placed on the perforated ceramic clapboard above the T-51 plates inside a desiccator. Two PDA plates were placed instead of T-51 as positive control (Bc-only). Tomatoes inoculated by 10 μL of milliQ water instead of *B. cinerea* were set with two plates of T-51 as negative control (T-only). There were three desiccators for each treatment in three replicates. The desiccators were individually sealed and placed at 20 °C in the dark for 7 d. The necrotic lesion on each tomato fruit was scored for disease severity using the numerical rating scale of 0–5: 0 indicated healthy; 1 and 2 indicated lesion areas without mycelia on the surface accounted for 1–25% and 26–50% of total area, respectively; 3, 4, and 5 indicated lesion areas along with a mass of mycelia on the surface accounted for 26–50%, 51–75%, and 76–100% of total area, respectively. The disease index of each treatment was calculated using the following formula:

(1) $$Disease\;index=100\times \overset{n}{\underset{i=0}{\sum }}\left(L_{n}\times n\right)/5\times \overset{n}{\underset{i=0}{\sum }}\left(L_{n}\right)$$

(where, *n*: the rating scale, *L_n_*: the number of the fruit corresponding to the rating scale *n*). This experiment was repeated twice.

### 2.4. Efficacy of VOCs of T-51 in Ggrowth Promotion of Arabidopsis Thaliana 

*A. thaliana* ecotype Col-0 was used in this study. The setup for exposure of *A. thaliana* plants to T-51 is performed in an airtight plant culture Magenta box. Twenty milliliters of PDA media were put into a 70 × 70 × 100 mm plant culture Magenta box, autoclave at 120 °C for 20 min. Before the PDA media solidified, four autoclaved flat bottom microtubes were made to stand in the box and fixed when the media cooled down. A mycelial plug of T-51 was inoculated in the center of PDA. A solution containing 2 mL of 0.2 × MS media (Murashige and Skoog media, Sigma Chemical Company, St. Louis, MO, USA, contains 1% agar) was added in each microtube. Col-0 seeds were surface sterilized using 50% ethanol for 1 min followed by 50% bleach for 10 min and washed thrice using autoclaved water [18,19] before placing one seed each on the surface of 0.2 × MS media in the microtubes. The box was closed tightly and cultured at 22 °C with 16 h photoperiod for four weeks. There were 10 boxes for T-51 treatment, and 10 boxes without T-51 as control. Diameter, fresh weight, and root length of each individual plant were measured. The chlorophyll content of each plant was extracted using 1 mL of 80% acetone at 4 °C in the dark and measured for absorbance at 663 and 645 nm. The total chlorophyll concentrations (chlorophyll a and b) were determined using the formula [3,20]:

Chlorophyll concentration = (8.02 × A663 + 20.2 × A645) × 1 mL/fresh weight(2)

This experiment was repeated twice. 

### 2.5. Analysis of VOCs of T-51 

The chemical components of VOCs of *T. koningiopsis* T-51 were analyzed using gas chromatography-mass spectrometry (GC-MS). T-51 was inoculated into a 250 mL flask containing 50 mL of PDA media, sealed using parafilm, and incubated at 20 °C for 5 d. The VOCs in the flask were collected using headspace solid-phase microextraction (SPME, divinylbenzene/carboxen/polydimethylsiloxane 50/30-μm SPME fiber, Sigma-Aldrich, St. Louis, MO, USA), the VOC components were identified using a GC-MS machine (Thermo scientific TRACE GC ΜLTRADSQ Π, San Jose, CA, USA) with a J & WHP-5MS fused-silica capillary column (30 m × 0.25 mm × 0.25 µm, Agilent Technologies Inc., Palo Alto, CA, USA). GC was operated in spitless mode with the injector temperature at 250 °C. The column temperature was programmed as follows: 40 °C for 3 min, then increased to 160 °C by 3 °C/min, 160 °C for 2 min, then increased to 220 °C by 8 °C/min, 220 °C for 3 min. Helium (ultra-high purity) was used as the carrier gas, which was set at a flow rate of 1.2 mL/min. MS was operated in electron ionization mode at 70 eV and 230 °C. Mass spectra were obtained using scan modus with the total ion counts within the range of 50 to 600 m/z. The VOCs released from the non-inoculated PDA were also collected and identified in the same manner as the control. This experiment was conducted two times. The VOCs appearing in the T-51 culture but not appearing in the control were considered to be the components produced by T-51. 

### 2.6. Statistical Data Analyses

Colony diameter data of *B. cinerea* treated with VOCs of different *Trichoderma* isolates as well as the tomato fruit disease index between different amounts of T-51 treatment and control were compared using Duncan’s multiple range test with significance level set at *p* < 0.05 using SAS program (version 8.1, SAS Institute, Cary, NC, USA). The colony diameter, conidia germination and germ tube length of *B. cinerea* and *F. oxysporum*, and the plant growth data of T-51 treatment and control were compared using student’s *t* test at *p* < 0.05 (*) or *p* < 0.01 (**).

## 3. Results

### 3.1. Inhibition of B. Cinerea and F. oxysporum by Trichoderma Isolates

**Figure 1:**
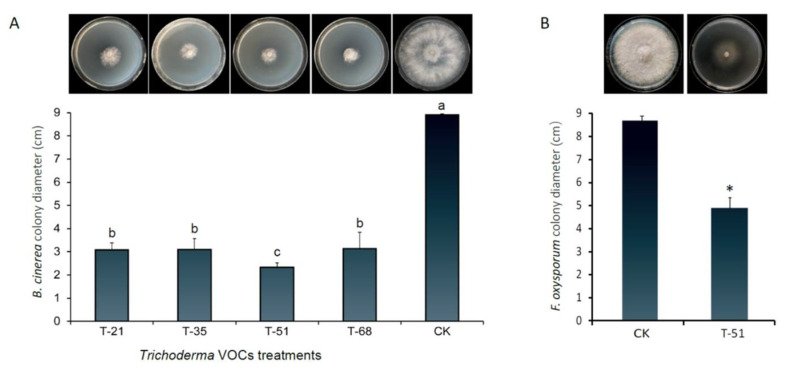
Effect of the volatile organic compounds produced by *Trichoderma* isolates on the mycelial growth of plant pathogenetic fungi *Botrytis cinerea* (**A**), and *Fusarium oxysporum* (**B**). CK was a negative control that was treated using PDA instead of T-51. Means ± SD (*n* = 3) labeled with the same letters in each histogram are not significantly different (*p* > 0.05) from each other according to least significant difference test. Means ± SD (*n* = 3) labeled with * are significantly different (*p* < 0.05) compared with the control according to student’s *t* test.

In the double-dish experiment, *B. cinerea* mycelial growth was evidently suppressed by the *Trichoderma* isolates T-21, T-35, T-51, and T-68 compared with that in the control, and the inhibition percentages were 65.45, 65.17, 73.78, and 64.79%, respectively. The inhibition activity of *T. koningiopsis* T-51 was significantly better than that of other isolates (Figure 1A). T-51 also showed strong inhibition (43.68%) of *F. oxysporum* mycelial growth by producing VOCs with 8.7 ± 0.2 cm and 4.9 ± 0.5 cm colony diameters of control and T-51 treatment, respectively (Figure 1B). Moreover, the colony of *F. oxysporum* was abnormal with fewer aerial hyphae and less spore production.

**Figure 2:**
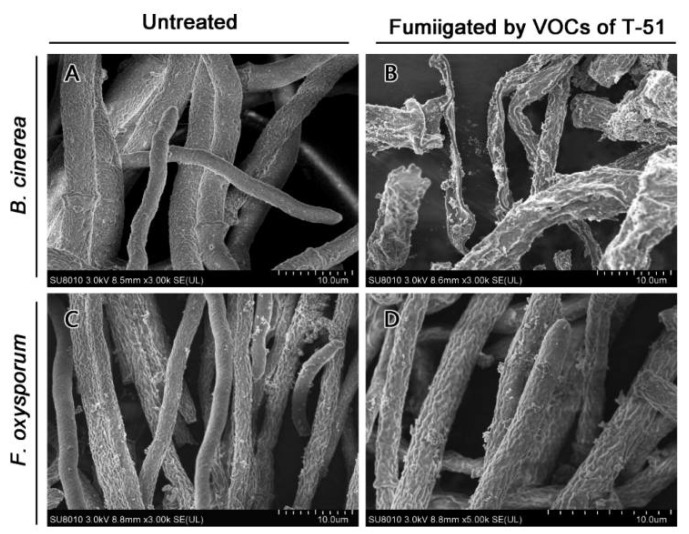
Scanning electron micrographs showing the hyphal morphology of *Botrytis cinerea* (**A**,**B**), and *Fusarium oxysporum* (**C**,**D**) fumigated by volatile organic compounds (VOCs) of T-51 (**B**,**D**), or untreated (**A**,**C**). A and C show the untreated healthy hyphae; (**B**) shows the shriveled hyphae of *B. cinerea* treated by T-51 VOCs.

SEM observation showed that the hyphae of untreated *B. cinerea* was vigorous and turgid (Figure 2A); however, the hyphae of *B. cinerea* fumigated by T-51 VOCs looked shriveled and collapsed (Figure 2B). Interestingly, *F. oxysporum* did not show much difference between the control and treatment (Figure 2C,D).

**Figure 3:**
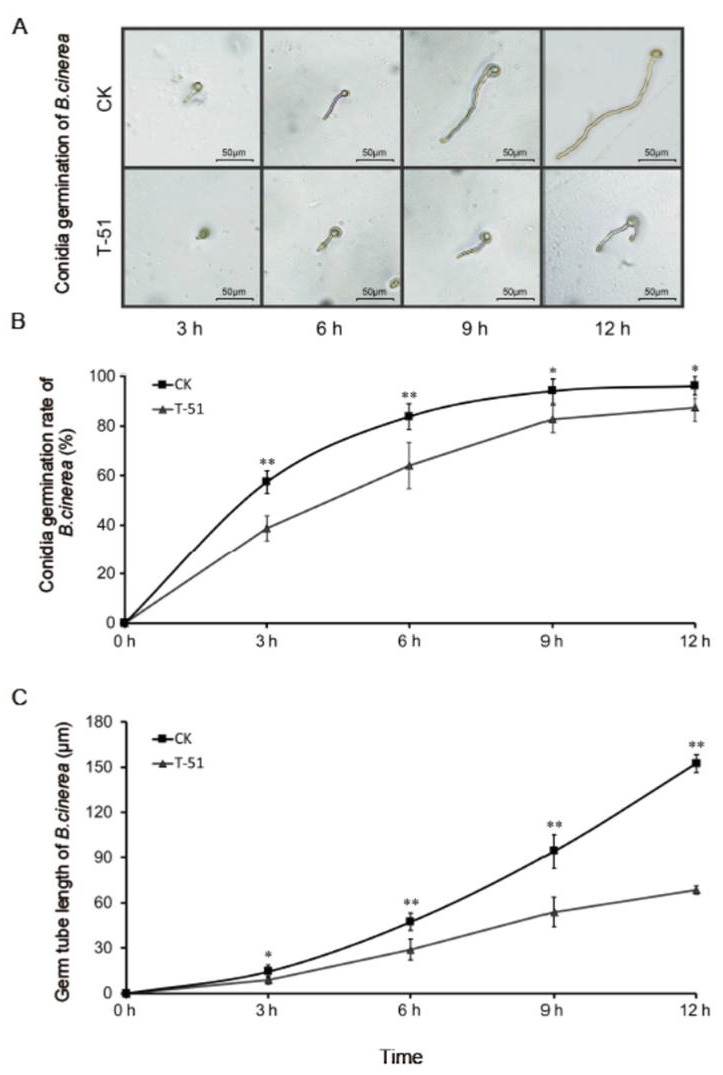
Effect of the volatile organic compounds produced by T-51 on the conidial germination of *Botrytis cinerea*. (**A**): *B. cinerea* conidia germination under microscope; (**B**): germination rate of *B. cinerea* conidia at 3 h, 6 h, 9 h, and 12 h; (**C**): germ tube length of germinated conidia of *B. cinerea* measured under microscope. Means ± SD labeled with * and ** are significantly different at *p* < 0.05 and *p* < 0.01, respectively, compared with the control at the same time point.

**Figure 4:**
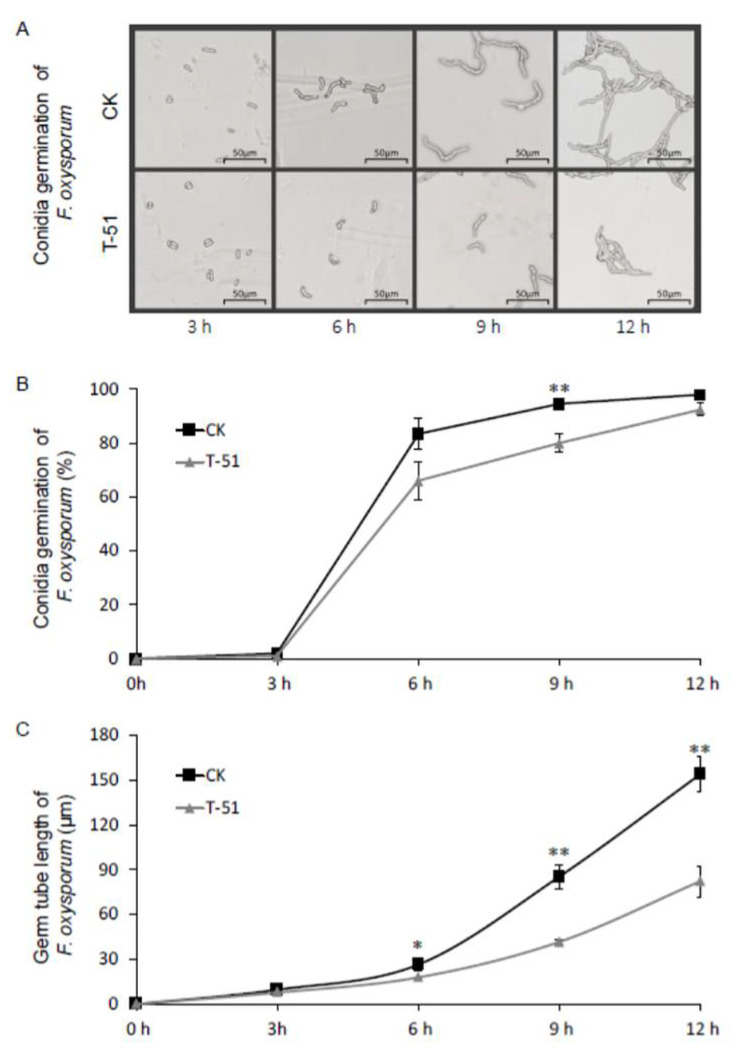
Effect of the volatile organic compounds produced by T-51 on the conidial germination of *Fusarium oxysporum*. (**A**): *F. oxysporum* conidial germination under microscope; (**B**): germination rate of *F. oxysporum* conidia at 3 h, 6 h, 9 h, and 12 h; (**C**): germ tube length of germinated conidia of *F. oxysporum* measured under microscope. Means ± SD labeled with * and ** are significantly different at *p* < 0.05 and *p* < 0.01, respectively, compared with the control at the same time point according to student’s *t* test.

The inhibition of *B. cinerea* and *F. oxysporum* conidial germination by T-51 VOCs was measured. The *B. cinerea* conidia in T-51 treatment showed greater suppressed germination compared to the control (Figure 3A). The results showed that the conidia in T-51 treatment germinated slower than the control, especially from 3 h to 9 h (Figure 3B). The germ tube elongation of *B. cinerea* was significantly suppressed by VOCs of T-51 from 3 h and the inhibition percentage at 12 h was 55.1% (Figure 3C). Similar results were observed on *F. oxysporum* conidial germination (Figure 4A), where germination under T-51 treatment was significantly slower; however, the final germination percentage did not show a significant difference compared with the control (Figure 4B). The germ tube elongation of *F. oxysporum* was also inhibited by VOCs of T-51 from 6 h (Figure 4C).

### 3.2. Efficacy of the VOCs of T-51 in Control of Botrytis Fruit Rot of Tomato

**Figure 5:**
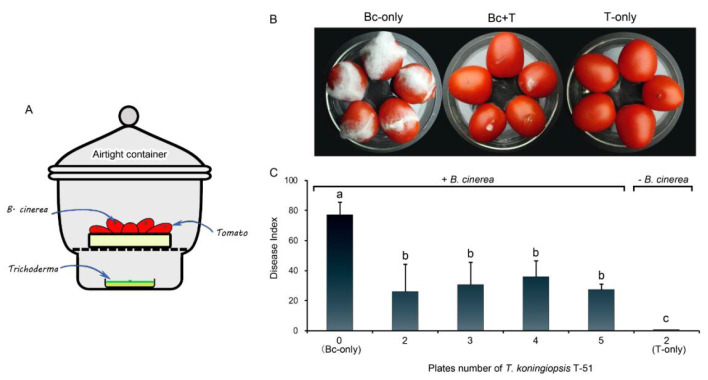
Efficacy of the volatile organic compounds (VOCs) produced by T-51 (T) on suppression of infection by *Botrytis cinerea* (Bc) on tomato fruits. (**A**): diagrammatic figure of the fruit infection assay. (**B**): The infection of *B. cinerea* on tomato fruits under different treatments. (**C**): Disease index of tomato fruits infected by *B. cinerea*; different number of T-51 plates indicate different VOCs produced in the treatment container. Means ± SD (*n* = 10) labeled with the same letters in each histogram are not significantly different (*p* > 0.05) from each other according to the least significant difference test.

The tomato fruit rot suppression experiment by the VOCs of T-51 was set in an airtight glass desiccator as shown in Figure 5A. After incubation at 20 °C or 7 days, all the tomato fruits in Bc-only control showed soft rot and mass of mycelia with a disease index reaching 77.3 (Figure 5B,C). However, in the presence of the T-51 culture plates, the fruit rot disease was significantly suppressed, with a disease index that decreased to 26.0, 30.7, 36.0, and 27.3 for 2, 3, 4, and 5 plates of T-51, respectively. There was no significant difference between the disease index and number of plates of T-51(Figure 5C). The wounded tomatoes treated with T-51 but not by *B. cinerea* (T-only) were still healthy (Figure 5B,C).

### 3.3. Efficacy of the VOCs of T-51 in Growth Promotion of A. Thaliana

**Figure 6:**
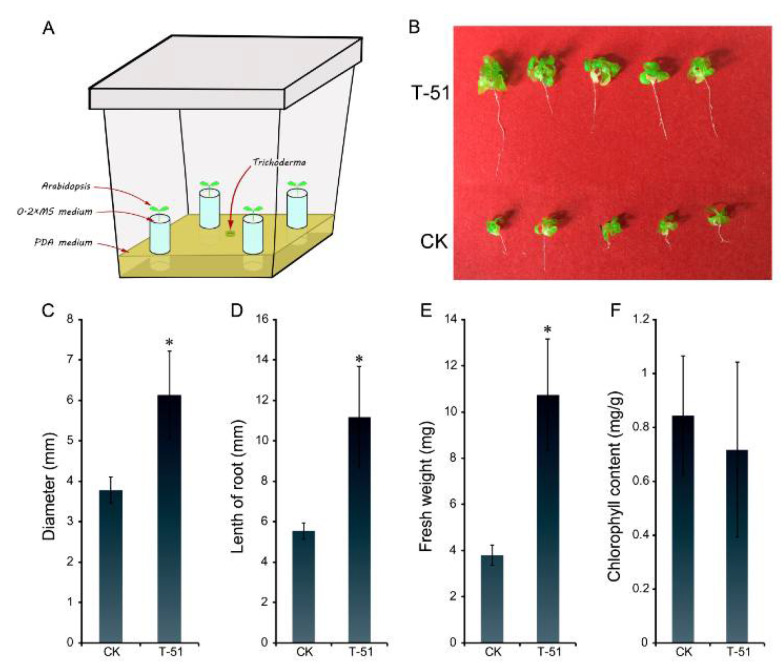
Efficacy of the volatile organic compounds (VOCs) produced by T-51 on promotion of *Arabidopsis thaliana* growth. (**A**): diagrammatic figure of the plant growth assay; (**B**): the promotion of *A. thaliana* growth by T-51 VOCs treated for 20 days. (**C**): Diameter; (**D**): length of root; (**E**): fresh weight; and (**F**): chlorophyll content of *A. thaliana*; CK was a control where *A. thaliana* was not treated by T-51. The * means significantly different *p* < 0.05 between control and T-51 according to student’s *t* test.

As seen in Figure 5A, the *A. thaliana* seedling exposed to T-51 VOCs showed better growth than the untreated control after 4 weeks (Figure 6B). The *A. thaliana* rosette leaves diameter, root length and fresh weight of the VOC-treated plants were significantly increased compared with the control (Figure 6C–E). The mean rosette diameter of *A. thaliana* treated by VOCs was 6.14 mm, which was a 62% promotion compared to control (3.78 mm); the mean root length in T-51 treatment was 11.17 mm, which is approximately 2-fold of the control (5.55 mm); and the fresh weight of T-51 treatment was 10.73 mg, which was approximately 3-fold of that in the control (3.81 mg). The chlorophyll contents were measured and did not show significant difference between the T-51 and control treatments (Figure 6F).

### 3.4. GC-MS Analysis of the VOCs of T. Koningiopsis T-51

GC-MS analysis identified 24 possible compounds (relative peak area (RA) > 0.1%) released from 5-day-old PDA culture of T-51 (Table 1). Most compounds were alkenes, alkanes, or esters. The most abundant compounds in this VOCs profile were β-phellandrene (retention time (RT): 11.02 min); 1,3,6,10-cyclotetradecatetraene,3,7,11-trimethyl-14-(1-methylethyl)-, [S-(E,Z,E,E)]- (RT: 47.06 min); oxime-, methoxy-phenyl- (RT: 6.62 min); cycloocta-2,4-dien-1-ol (RT: 44.62 min); and oxacyclododec-9-en-2-one, 2-methyl-, (E)- (RT: 48.38 min). The RAs were 7.22, 6.88, 5.32, 4.88, and 4.00, respectively. 

## 4. Discussion

VOCs produced by fungi play a critical role in ecological fitness due to their low molecular weight, and they vaporize at normal temperatures and pressures. The function of fungal VOCs has been concluded by Li et al. [21] as chemical tools against other microbes, signals for intra- and inter-species communications, and regulators of plant growth and/or stress resistance. In plant disease biological control process, biological control agents such as *Trichoderma* spp. are not always in direct contact with the pathogen or plant; however, VOCs could be complementary in the indirect interactions between a biological control agent (BCA) and plant pathogenic fungi or between BCA and plant. *T. koningiopsis* T-51 has been reported to have potential for biologically controlling *B. cinerea* and promoting growth in tomato plants [14] by direct contact with pathogen or plant. In this study, VOCs of T-51 showed inhibition of *B. cinerea* infection on tomato fruit, indicating another possible mechanism and application method in T-51 biocontrol.

VOCs from microbes have been reported to have antifungal activity against plant pathogenic fungi such as *B. cinerea* and *F. oxysporum*. For example, *T. koningiopsis* T-403 VOCs showed better antifungal activity against ginseng-root rot fungi compared with fungicides mancozeb (20 μg/mL) or fenhexamid (20 μg/mL) [22]; the VOCs from *Candida intermedia* strain C410 showed suppression of conidial germination and mycelial growth of *B. cinerea* and control of Botrytis fruit rot of strawberry [16]; VOCs produced by *F. oxysporum* CanR-46 were highly inhibitory to *B. cinerea* mycelial growth and infection on tomato [23]; VOCs produced by BCA *Bacillus amyloliquefaciens* NJN-6 showed inhibition of the growth and spore germination of *F. oxysporum* f. sp. *cubense* [24]; VOCs produced by *Paenibacillus polymyxa* WR-2 inhibited the growth and spore germination of *F. oxysporum* f. sp *niveum* [25]. Four *Diaporthe* spp. Isolates, which were endophytic fungi in *Catharanthus roseus*, produced volatile metabolites showing antifungal properties against various plant pathogenic fungi such as *Alternaria alternata, B. cinerea, Colletotrichum gloeosporioides, F. graminearum,* and *Phytophthora cinnamomi* [26]. This study revealed that the VOCs produced by *T. koningiopsis* T-51 highly inhibited mycelial growth, conidial germination, and germ tube elongation of *B. cinerea* and *F. oxysporum*.

T-51 has been reported to have plant growth promotion ability when inoculated on roots in a previous study [14]. However, in this study, T-51 showed plant growth promotion without physical contact with the plant, through volatile compounds. Jalali et al. [27] reported that *T. koningiopsis* and other *Trichoderma* spp. isolates promoted *A. thaliana* growth and salt stress tolerance by VOCs. Lee et al. [28] showed that the VOCs of some *Trichoderma* spp. strains such as *T. viride*, and *T. pseudokoningii* promoted growth of *A. thaliana* but some *Trichoderma* strains could not, suggesting that the plant growth promotion by VOCs differs between species and isolates. Volatile compound cedrene produced by plant beneficial *T. guizhouense* NJZU4742 showed stimulation of plant growth and root development through regulated auxin signal transportation [29]. *Trichoderma* VOCs, in many plant experiments were processed in partitioned Petri dishes. In our study, we designed a new installation for the plant growth experiment as the growth of T-51 was too vigorous and contaminated the plant growth media in partitioned Petri dish.

The VOCs of T-51 were identified as alkenes, alkanes, and esters using GC-MS. Some of these compounds have been reported in other *Trichoderma* isolates. For example, β-phellandrene and phenylethyl alcohol were also detected in VOCs of *T. atroviride* ATCC 74,058 [30], and benzothiazole was detected in VOCs of *T. asperelloides* TSU1 [31]. Chen et al. [32] reported that the VOCs produced by a *T. koningiopsis* isolated YIM PH30002 showed antifungal activity against plant pathogen *Epicoccum nigrum* and detected β-phellandrene, cyclohexene, and cycloocta-2,4-dien-1-ol, which were also detected in VOCs of T-51 in our study. Phenylethyl Alcohol (PEA), also called 2-phenylethanal, was detected in a variety of plants and microbes, as well as the VOCs of T-51. PEA showed antifungal activity against *Candida* spp. [33] and *Penicillium* spp. [34]. PEA treated strawberry showed a significant decrease in the postharvest gray mold disease when it was inoculated by *B. cinerea* at 4 °C [35]. In this study, PEA in the VOCs of T-51 might play a role in strongly inhibiting against *B. cinerea* on tomato.

It is important for *Trichoderma* spp. to have multiple biocontrol mechanisms when being used for plant disease control or plant growth promotion in different situation. In this study, the efficacy of VOCs produced by *T. koningiopsis* T-51 showed strong growth inhibition of two plant pathogenic fungi, and further decreased the gray mold disease on tomato fruit. On the other hand, the VOCs of T-51 showed a promotion of plant growth. These indicate that VOCs probably work in the biocontrol process of T-51 before it directly becomes a mycoparasite in plant pathogenic fungi or colonizes the plant.

## Figures and Tables

**Table 1:** Volatile organic compounds produced by *Trichoderma koningiopsis* T-51 on potato dextrose agar plate at 5 days, as detected via gas chromatography–mass spectrometry analysis.

RT (min)	RA (%)	Compound Name	Cas #
4.53	2.39	Cyclotrisiloxane, hexamethyl-	541-05-9
6.62	5.32	Oxime-, methoxy-phenyl-	NA
10.15	2.49	Cyclotetrasiloxane, octamethyl-	556-67-2
11.02	7.22	β-Phellandrene	555-10-2
11.67	0.69	Benzeneacetaldehyde	122-78-1
14.69	1.49	Phenylethyl Alcohol	60-12-8
17.03	0.80	Cyclopentasiloxane, decamethyl-	541-02-6
19.44	0.20	Benzothiazole	95-16-9
20.61	0.50	Cyclotetrasiloxane, octamethyl-	556-67-2
24.44	0.11	Ethanone, 1-(3,4-dimethylphenyl)-	3637-01-2
24.66	0.88	Cyclohexasiloxane, dodecamethyl-	540-97-6
27.05	1.04	Cyclopentasiloxane, decamethyl-	541-02-6
27.39	2.33	Tetradecane	629-59-4
29.70	1.37	Caryophyllene	87-44-5
30.68	1.91	Benzene, 1-(1,5-dimethyl-4-hexenyl)-4-methyl-	644-30-4
31.46	0.69	Tetradecane, 2,6,10-trimethyl-	14905-56-7
31.77	3.36	Cycloheptasiloxane, tetradecamethyl-	107-50-6
38.21	1.76	Cyclooctasiloxane, hexadecamethyl-	556-68-3
39.24	0.39	Tetradecane, 2,6,10-trimethyl-	14905-56-7
44.62	4.88	Cycloocta-2,4-dien-1-ol	NA
46.69	0.18	(R,1E,5E,9E)-1,5,9-Trimethyl-12-(prop-1-en-2-yl)cyclotetradeca-1,5,9-triene	31570-39-5
47.06	6.88	1,3,6,10-Cyclotetradecatetraene, 3,7,11-trimethyl-14-(1-methylethyl)-, [S-(E,Z,E,E)]-	1898-13-1
47.73	2.51	(S,E)-8,12,15,15-Tetramethyl-4-methylenebicyclo [9.3.1]pentadeca-7,11-diene	386223-19-4
48.38	4.00	Oxacyclododec-9-en-2-one, 12-methyl-, (E)-	33644-08-5

RT: retention time, RA: relative peak area, VOCs of T-51 with the relative peak area less than 0.1% are not included in this table.
